# Levy Sooty Tern Optimization Algorithm Builds DNA Storage Coding Sets for Random Access

**DOI:** 10.3390/e26090778

**Published:** 2024-09-11

**Authors:** Jianxia Zhang

**Affiliations:** 1 College of Mathematics and Information Science, Henan Normal University, Xinxiang 453003, China; zhangjianxia@hait.edu.cn; 2 School of Intelligent Engineering, Henan Institute of Technology, Xinxiang 453003, China

**Keywords:** DNA storage, DNA coding, levy sooty tern optimization algorithm, random access

## Abstract

DNA molecules, as a storage medium, possess unique advantages. Not only does DNA storage exhibit significantly higher storage density compared to electromagnetic storage media, but it also features low energy consumption and extremely long storage times. However, the integration of DNA storage into daily life remains distant due to challenges such as low storage density, high latency, and inevitable errors during the storage process. Therefore, this paper proposes constructing a DNA storage coding set based on the Levy Sooty Tern Optimization Algorithm (LSTOA) to achieve an efficient random-access DNA storage system. Firstly, addressing the slow iteration speed and susceptibility to local optima of the Sooty Tern Optimization Algorithm (STOA), this paper introduces Levy flight operations and propose the LSTOA. Secondly, utilizing the LSTOA, this paper constructs a DNA storage encoding set to facilitate random access while meeting combinatorial constraints. To demonstrate the coding performance of the LSTOA, this paper consists of analyses on 13 benchmark test functions, showcasing its superior performance. Furthermore, under the same combinatorial constraints, the LSTOA constructs larger DNA storage coding sets, effectively reducing the read–write latency and error rate of DNA storage.

## 1. Introduction

DNA molecules are a natural storage medium, having been carriers of life for thousands of years. DNA molecules can also preserve non-biological information [1,2,3,4]. Since the validation of scalable DNA storage by the George Church team [5] at Harvard University in 2012, research related to DNA storage has increased annually. The first step in DNA storage is encoding information into DNA sequences [3,6]; encoding algorithms based on basic mapping relationships is relatively simple but sacrifices a certain degree of coding information density [7]. In 2012, Church et al. [5] mapped a draft of an HTML-encoded book into DNA as a 5.27 MB bit stream using a binary-to-base mapping method. The DNA-coding method developed by the Church team propelled research and applications in this field. In 2013, Nick Goldman proposed [8] a ternary information transformation model. Firstly, the Huffman ternary tree is used to analyze the appearance frequency of single bytes in binary files to be transcoded, converting binary sequences (0/1) into corresponding ternary sequences (0/1/2). Subsequently, based on the ternary mapping pattern, the current base of the DNA sequence is determined, as well as the previously selected base. This method completely avoids the repetition of consecutive bases but cannot regulate the GC content under fixed rules and may result in fragment repetition. The Goldman code was the first to introduce Huffman coding into DNA storage and was the first coding method to consider base information density. Due to the complexity of DNA synthesis and sequencing processes involving many intricate experimental operations [9,10,11], chemical reactions [12], and unavoidable noise pollution [13], unpredictable DNA-specific errors can occur at any time, leading to base loss, incorrect connections, or other unexpected changes, resulting in erroneous DNA sequences.

To reduce the error rate, Grass et al. [14] introduced error correction codes from information and communication technology into DNA storage, utilizing Reed–Solomon (RS) codes to rectify base errors or sequence losses occurring during DNA storage. The theoretical information density of the Grass et al. coding method can reach 1.78 bits per nucleotide (nt), marking the first integration of error correction algorithms into DNA coding processes and expanding the encoding module of DNA storage. In 2016, the Meinolf Blawat team [15] developed an efficient and robust forward error correction scheme for DNA channels by using bytes as the basic unit of base conversion and mapping eight bits of information into five nucleotides. The remaining two bits represented optional conversion parts. This design restricted the maximum length of homopolymers to three, reducing the likelihood of DNA sequence self-complementarity. Phylogenetic analysis methods [16] that use unique natural vectors to represent DNA sequences also help to ensure one-to-one correspondence in DNA storage, which ensures that each DNA sequence is represented clearly and unambiguously in genome space. In 2017, Yaniv Erlich and Dina Zielinski [17] proposed a coding algorithm based on the Luby Transform, unprecedentedly boosting the encoding rate to 1.98 bits/nt. In 2020, Press et al. [18] developed the Hash Encoded, Decoded by Greedy Exhaustive Search (HEDGES) DNA encoding algorithm capable of handling insertion and deletion errors in DNA synthesis and sequencing errors. This algorithm employs RS codes and convolution codes for encoding. The results show that, while sacrificing a certain encoding density, HEDGES can handle approximately 1.2% of insertion and deletion errors. Cai et al. [19] emphasize the importance of redundancy and error correction to maintain the uniqueness of the encoded DNA sequences and to ensure that the original data can be retrieved accurately even if errors occur.

The random access process of DNA storage based on Polymerase Chain Reaction (PCR) first requires the design of a specific primer library to ensure the uniqueness of the random access target. The number of fixed-length primers is limited, and increasing the primer length to increase the number of primers will result in a decrease in the bases available for data in the DNA sequence [1,20]. In 2018, Organick et al. [21] proposed an encoding algorithm that significantly reduces sequencing redundancy by random access, thereby requiring fewer physical copies of given molecules to fully recover stored data. Moreover, the random-access DNA storage system can also represent file metadata by impervious silica capsules selecting barcodes [22], enabling the completion of Boolean logical searches without the use of methods. Anavy et al. [23] encoded binary using a six-letter composite DNA alphabet and combined RS and fountain codes for error correction. The information stored in DNA is converted from standard American Standard Code for Information Interchange (ASCII) [23] encoding to binary sequences, and Huffman coding is used to generate DNA sequences. In 2023, Yu et al. [24] overcame the problem of the passive processing of DNA storage data in DNA pools by realizing an active DNA data-editing process in a droplet-controlled jet (DCF) system using splint connections.

To reduce inherent errors in the random-access DNA storage process, Cao et al. [25] reported new combination constraints and proposed a Damping Multi-Verse Optimizer (DMVO) algorithm to design DNA storage encoding sets that meet combination constraints, using these encodings as address bits. Based on this, they proposed a thermodynamic Minimum Free Energy (MFE) constraint [26] for the construction of DNA storage coding sets. The MFE constraint is used to avoid nonspecific hybridization and reduce synthesis sequencing error rates, and a new BMVO algorithm is used in this work. Yin et al. [27] proposed the NOL-HHO algorithm by improving the Harris Hawks optimization algorithm, which achieves a better lower bound for DNA storage coding. Although the results of the NOL-HHO algorithm are improved in the work of Limbachiya et al., there is still much room for improvement in its lower bound. In 2022, Rasool et al. [28] proposed a new DNA data storage (BO-DNA) biological optimization coding model to overcome reliability issues.

Although the construction of DNA storage coding sets can be equivalent to the optimization problem of satisfying combination constraints, existing encoding algorithms still have deficiencies in the quantity and quality of encoding. Therefore, this paper introduces Levy flight operations to improve the STOA and proposes the LSTOA, reducing the likelihood of the original algorithm falling into local optima and accelerating convergence speed. The encoding results show that under the same combination of constraint conditions, the LSTOA can construct larger DNA storage coding sets, providing more address bits for random access to reduce DNA storage read–write latency.

## 2. Coding Constraints

### 2.1. GC Content Constraint

In DNA sequences, the bases A, T, C, and G form bonds with each other, but the bonds between the bases can vary. Therefore, during DNA storage, it is desirable to achieve a balanced ratio between the bases, especially between AT and GC. This is because GC bonds have three hydrogen bonds, which can make the DNA sequence more stable. Generally, in DNA sequence design, a GC content of 40–60% is recommended [29], and *GC(x)* can be calculated using the following formula for a DNA sequence *x*.

(1) $$GC(x)=\frac{\left|G|+|C\right|}{\left|x\right|}\times 100\%$$

### 2.2. Edit Distance Constraint

In DNA storage, the hamming distance can measure the similarity between two codewords. However, it does not take into account the positional advantage of DNA sequences in solution. Therefore, this paper introduce the edit distance constraint to overcome this limitation [25]. Because edit distance measures the minimum number of operations (including insertion, deletion, and substitution operations) by which sequences are interconverted, this distance metric is well suited for DNA storage. This is because it takes into account sequence changes in solution, especially common errors such as insertions, deletions, and substitutions in nanopore sequencing. Considering this, the edit distance constraint can also partially correct errors in DNA nanopore sequencing.

The definition of edit distance in DNA storage is as follows: for DNA codewords *m* and *n* of length *l*, *S*(*m*, *n*) is the edit distance between *m* and *n*. Therefore, the edit distance constraint is denoted as *ME*(*u*), where *ME* is the minimum value of *S* and *d* is minimum edit distance.

(2) $$ME(u_{i})=\underset{i\leq j\leq n,i\neq j}{\min}\{S(m_{i},n_{i})\}\geq d$$

### 2.3. No-Runlength Constraint

Consecutive bases can lead to DNA sequence instability and potentially increase the error rate during synthesis and sequencing. Therefore, it is necessary to consider the impact of continuous bases on DNA storage encoding. The use of a No-runlength constraint [9] can effectively mitigate this issue. The No-runlength constraint is defined as follows: for a DNA codeword *L* (*l*_1_, *l*_2_, *l*_3_… *l_n_*) of length *n*, it can be calculated using the following formula:

(3) $$L_{i}\neq L_{i+1}\;\;\;\;\;\;\;\;i\in [1,\;n-1]$$

For example, in “TAAAATCG”, “A” is repeated, making it easy to misread long “A” as short “A” during synthesis and sequencing. This increases the error rate in DNA storage information and decreases the read–write coverage.

## 3. Algorithm Description

### 3.1. Sooty Tern Optimization Algorithm

The STOA [30] is a biologically inspired optimization algorithm that simulates the migration and attack behaviors of real-life sooty terns. When dealing with constrained optimization problems, the STOA employs a static penalty method to handle the constraints. In this method, a penalty value is assigned to each infeasible solution. This penalty function method helps convert constrained optimization problems into unconstrained ones, making it easier for the search process to find feasible domains. Here, the main principles of the STOA are listed.

Firstly, there is the migration behavior, representing the global search process, where search agents (sooty terns) in the STOA change their speed and attack angles during migration. They increase altitude using their wings and follow the following formula to avoid collisions:

(4) $$\vec{C_{st}}=S_{A}\times \vec{P_{st}}(z)$$

P_st_ is the old position, *z* is the iteration number, and *S_A_* is updated as follows:

(5) $$\begin{array}{l} S_{A}=C_{f}-(z\times (C_{f}/Max_{iterations}))\\ where,z=0,1,2,\ldots ,Max_{iterations} \end{array}$$

After updating the position, the current individual approaches the optimal individual to obtain a better position:

(6) $$\vec{M_{st}}=C_{B}\times (\vec{P_{bst}}(z)-\vec{P_{st}}(z))$$

$\vec{M_{st}}$ is the relative distance between the current individual and the optimal individual, $\vec{P_{bst}}$ is the position of the current optimal individual, and *C_B_* is calculated as follows:

(7) $$C_{B}=0.5\times R_{and}$$

where R_and_ is a random number in the range of [0, 1]. Once collision-free positions and relative distances are determined, the next step is to approach the optimal individual:

(8) $$\vec{D_{st}}=\vec{C_{st}}+\vec{M_{st}}$$

During the attack process, sooty terns generate spiral behavior, described as

(9) $$x^{\prime }=R_{adius}\times \sin(i)$$

(10) $$y^{\prime }=R_{adius}\times \cos(i)$$

(11) $$z^{\prime }=R_{adius}\times i$$

(12) $$r=u\times e^{kv}$$

where R_adius_ is the radius of spiral flight, u = v = 1 defines the spiral shape, and *i* is the spiral angle in [0, 2*π]. During the optimization process, search agents converge towards the direction of the best neighbor for position updates.

(13) $$\vec{P_{st}}(z)=(\vec{D_{st}}\times (x^{\prime }+y^{\prime }+z^{\prime }))\times \vec{P_{bst}}(z)$$

### 3.2. Levy Sooty Tern Optimization

Levy flight is a random walk where the step lengths follow a heavy-tailed probability distribution [31]. This strategy is used in optimization and search algorithms, particularly in scenarios where the search space is complex and high-dimensional. Levy flight helps the algorithm escape local optima by occasionally taking large steps, which allows it to explore new regions of the search space. The heavy-tailed nature of the step distribution means that while most steps are small, some are large, leading to a balance between exploration and exploitation.

In a Levy flight, the step length *l* is drawn from a Levy distribution, which has a power-law tail:

(14) $$P(l)∼{\left|l\right|}^{-(1+\beta )},0<\beta \leq 2$$

P(l) is the Probability Density Function for the step *l*. This function describes the probability of occurrence of different step lengths *l*. In Levy flights, the step length usually obeys a heavy-tailed distribution, meaning that most of the steps are short and occasionally longer ones occur. β is the exponent that controls the distribution and determines how heavy-tailed the Levy distribution is. Typically, the value of β lies between 0 and 2 (0 < *β* ≤ 2). As β gets closer to 1, the distribution becomes more heavily tailed, i.e., the probability of a long step occurring is higher.

The STOA exhibits good exploratory abilities on multimodal and fixed-dimensional multimodal test functions, aiding in avoiding local optima. By simulating the migration and attack behaviors of real-life sooty terns, it aims to find the best solution during optimization. It demonstrates good exploration and exploitation capabilities when dealing with different types of test functions, enabling it to find optimal solutions in complex optimization problems. However, the STOA may converge to local optima prematurely. Although it possesses good exploratory and exploitative capabilities, its performance may be influenced by initial conditions and random factors, leading to insufficient stability. Hence, this paper introduces the Levy flight strategy to replace the globally optimal update strategy in the STOA [29], mitigating the impact of initial conditions and random factors on optimization results. Levy flight strategy is employed in the later iterations of the STOA for Levy flight operations, expanding the search range of the STOA and obtaining a larger code set.

### 3.3. Benchmark Function

In order to better illustrate the performance of the LSTOA, this paper introduces 13 benchmark test functions, whose expressions are as follows (Table 1 and Table 2). F1 (Sphere Function): The simplest test function where all local optima are near the global optimum. It is unimodal and is used to test the local search capabilities of algorithms. It is suitable for basic optimization algorithm performance testing. F2 (Schwefel’s Problem 2.22): It features a sharp global minimum point and a flat search space, challenging the algorithm’s global search capabilities. F3 (Elliptic Function): Scales differently across dimensions to test whether the algorithm can handle problems with different scales. F4 (Schwefel’s Problem 1.2): Features an asymmetric valley to test whether the algorithm can handle asymmetric optimization problems. F5 (Schwefel’s Problem 2.21): Features multiple local minima but only one global minimum to test the algorithm’s local avoidance capabilities. F6 (Rosenbrock’s Valley): Also known as the "banana function", it has a narrow, winding valley with the global minimum at one end, testing the algorithm’s path-tracking ability. F7 (Step Function): It tests whether the algorithm can handle discontinuous search spaces due to its discrete nature.

For high-dimensional multi-peak functions F8–F13: F8 (Quartic Noise Function): Introduces random noise to test whether the algorithm can handle noise effects. F9 (Schwefel’s Problem 2.26): A multimodal function with many local minima, used to test the algorithm’s global search capabilities and avoidance of local optima. F10 (Ackley’s Function): A complex multimodal function with the global minimum surrounded by many local minima, suitable for testing the algorithm’s global search capabilities. F11 (Griewank’s Function): Contains multiple regular local minima to test whether the algorithm can escape from these local minima. F12 (Rastrigin’s Function): A typical test problem with numerous local minima, used to test the algorithm’s performance in highly oscillating landscapes. F13 (Non-continuous Rastrigin’s Function): A non-continuous version of the Rastrigin function, increasing the difficulty of handling non-continuous problems. These 13 functions reflect most real-world problems, and testing them can effectively reflect the performance of algorithms. To ensure fairness, reliability of results, and rigor in the experiments, I limited the definition domain and number of iterations for the test functions.

## 4. Result and Analysis

### 4.1. Algorithm Performance Comparison

In this section, I selected 13 CEC benchmark functions. The CEC benchmark functions are regularly published by the IEEE CEC and are well known for the performance analysis of heuristic algorithms. The CEC benchmark functions are used to test and compare the summed performance of the different heuristic class algorithms, including the convergence speed, optimality finding ability, ability to jump out of local optimum, and exploration ability. In this paper, the single-peak function, multi-peak function, and hybrid function are selected to comprehensively analyze the performance of the LSTOA. The STOA is a pre-improvement algorithm, so the comparison is necessary. In addition, PSO emphasizes group intelligence and social learning, GA focuses on genetic evolution and stochastic search, SSA uses sparrow foraging and vigilance behaviors to balance exploration and exploitation, GWO snatches the leader’s guidance and other individuals’ following, and GSA is an algorithm based on the physical mechanism to know the searching process, and these diversities can help to comprehensively evaluate the performance of the new algorithm. All algorithms were run 30 times on the same functions, and the results are shown in Table 3 and Table 4.

F1–F7 are high-dimensional unimodal functions, focusing on the algorithm’s ability to find the global optimum, as well as the speed and stability of convergence to the global optimum. F8–F13 are high-dimensional multimodal functions, focusing on the algorithm’s ability to explore different regions, strategies to avoid local optima, and performance in facing complex search spaces. Table 3 and Table 4 display the performance of the LSTOA on the 13 test functions. In most cases, the LSTOA achieved competitive results. Additionally, from Table 4, it can be observed that LSTOA has smaller result variances, indicating greater stability. However, when facing complex functions such as F10 and F13, the performance of the LSTOA is not satisfactory. This may be due to the limitations of the Levy algorithm when dealing with multimodal functions, hence not achieving the optimal solution.

### 4.2. DNA Storage Code Set

In a random-access DNA storage system, different positions require different types of encoding. For data bits, also known as payload bits, encoding methods such as fountain codes are typically used to convert binary data into redundant DNA sequences. However, due to the shorter length and stricter constraints needed for addressing, address bits are not suitable for more complex encoding algorithms. To ensure the simplicity and robustness of the encoding process, this paper employs a heuristic algorithm to search for candidate encoding sets that meet the necessary constraints, thereby enabling the encoding of address bits in a random-access DNA storage system. Additionally, the encoding set constructed by the LSTOA can also carry data by designing a mapping dictionary between DNA codewords and binary information.

The DNA coding set with length *n*, distance *d*, GC content weight *w*, and satisfying editor distance constraint, GC content constraint, No-runlength constraint, and uncorrelated address constraint [33] is defined as $A^{GC,NL,UA}(n,d,w)$. The size of the coding set is illustrated in Table 5, where 4 *≤ n ≤* 9, 3 *≤ d ≤ n*. I model this multi-objective constrained optimization problem and propose an optimization objective function for optimization by the LSTOA.

(15) $$Function_{obj}=\sum_{i=1}^{n}H(s,s_{i})$$

where *H* is the storage edit distance function.

In order to further illustrate the optimization performance of the LSTOA, the size of set A is compared in this section, and the results are shown in Table 5. The comparison objects are Li and DMVO, which are the latest proposed DNA storage coding set design method and the most classical method, respectively. In Table 5, the union of previous optimal results are shown with a superscript *b*, where the superscript *l* is the result of this paper and the bolded results represent the optimal results under the same conditions. It can be seen that the LSTOA achieves ideal results in most cases. Especially when *n* = 8 and *d* = 3, the DNA storage coding set constructed by the LSTOA is 8.6% larger than the previous optimal result. This may be because the STOA may converge prematurely to a locally optimal solution. Although the STOA has good exploration and utilization abilities, it may converge to the local optimal solution prematurely and fail to find the global optimal solution for DNA storage and coding problems. The Levy algorithm solves this problem and uses the Levy flight strategy to jump out of the local optimal solution. When the DMVO algorithm is dealing with large-scale problems, the computational complexity of this multi-verse algorithm may be very high. Due to the need to search across multiple universes, and possibly a lot of iteration and computation, this can lead to long running times for the algorithm. The LSTOA runs faster, and renders it easier to find the approximate optimal solution in DNA storage coding problems.

Although LSTOA finds the current optimal in most cases, it expands the DNA storage coding set. However, in some cases, the set size is smaller than that of previous methods because the convergence rate of the STOA may be unstable, depending on the choice of initial solution, the setting of parameters, and the complexity of the problem. In some cases, the algorithm may converge quickly, while in others, it may take more iterations to converge to a satisfactory solution. To solve this problem, this paper added a Levy flight operation in the late convergence of the LSTOA to make it jump out of the current optimal and accelerate the convergence speed [34]. This can help Sooty Tern to converge faster and find the approximate optimal solution. This paper also analyzes the coding time, as shown in Table 6. Under the same constraints, a larger coding set can be constructed in less time due to the reduced computational complexity of the LSTOA using the Levy flight strategy.

**Figure 1:**
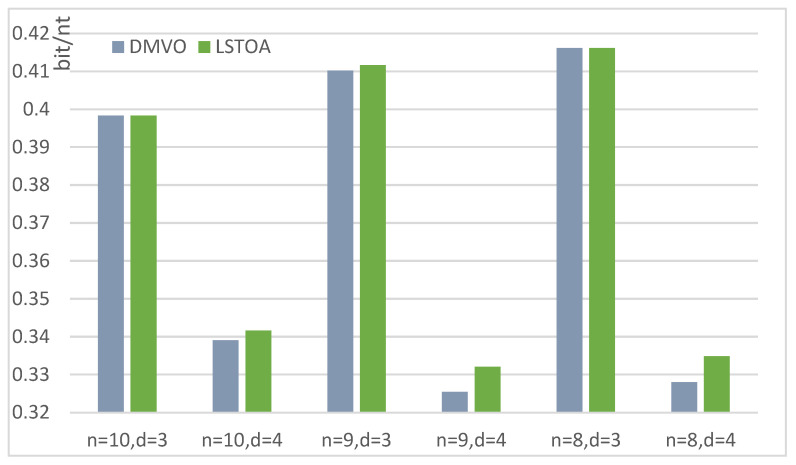
Comparison of DNA storage code rates for LSTOA and DMVO.

The improved LSTOA in this paper constructs a larger DNA coding set under the same constraints, which can not only effectively improve random access efficiency, but also increase the storage density. The code rate can be calculated by $R=\frac{\log_{4}M}{n}$, where *M* is the size of the coding set and *n* is the length of the codewords. In Figure 1, I compare the coding rates of the LSTOA and the previous optimal work DMVO when *M* is 8, 9, and 10. The x-axis is the different coding conditions, and y-axis is the size of the coding rate. The results in the table demonstrate the potential of the LSTOA in coding, especially at *d* = 4, where LSTOA increases the code rate by more than 1 percentage point.

## 5. Conclusions

In this paper, the LSTOA is proposed based on the Levy flight strategy to address the issue of local optima frequently encountered by traditional heuristic algorithms in optimization problems. To further illustrate the LSTOA, 13 benchmark test functions are introduced, some of which are high-dimensional unimodal functions for general testing purposes, while others are high-dimensional multimodal functions for extreme function performance testing. In these functions, the LSTOA achieved satisfactory results. In the practical problem of DNA storage encoding, the LSTOA addresses the issue of low encoding efficiency during DNA encoding. To enhance encoding quality, an editor distance constraint, GC content constraint, No-runlength constraint, and uncorrelated address constraint are introduced. The combined constraints can reduce errors in DNA storage and improve DNA storage efficiency, but also pose challenges to encoding and may reduce storage density. Therefore, by transforming the DNA storage encoding problem into a heuristic algorithm for solving multi-objective optimization problems, I iteratively generate DNA storage encoding sets that meet the constraints. Table 5 shows that the LSTOA expands the DNA storage encoding sets in four scenarios, and achieves similar results to previous studies in other scenarios. Cases such as *n* = 9, *d* = 3 indicate the potential of the LSTOA to surpass the existing DNA storage encoding set size, enabling better random access processes to access more data with the same codeword length. In other cases, although the DNA storage encoding sets constructed by the LSTOA do not expand, they remain consistent with the optimal results of previous studies, demonstrating the stability of the LSTOA. In Figure 1, I also compare the code rate, where a higher code rate indicates that more information can be stored using the same DNA sequence. Even a 1% improvement is significant for expensive DNA storage systems.

In future work, I will continue to focus on DNA storage encoding because encoding is crucial not only for the DNA storage data writing process but also for data reading. Clustering [35], assembly, and other processes require encoding, so considering setting clustering and assembly or other preset information in the encoding process may be a direction for our continued efforts.

## Figures and Tables

**Table 1:** Unimodal benchmark functions.

Function	Dim	Range	F_min_
$F_{1}(x)=\sum_{i=1}^{n}X_{i}^{2}$	50	[−100,100]	0
$F_{2}(x)=\sum_{i=1}^{n}\left|x_{i}\right|+\prod_{i=1}^{n}\left|x_{i}\right|$	50	[−10,10]	0
$F_{3}(x)=\sum_{i=1}^{n}{\left(\sum_{j-1}^{i}x_{j}\right)}^{2}$	50	[−100,100]	0
$F_{4}(x)=\max_{i}\{|x_{i}|,1\leq i\leq n\}$	50	[−100,100]	0
$F_{5}(x)=\sum_{i=1}^{n-1}[100{\left(x_{i+1}-x_{i}^{2}\right)}^{2}+{\left(x_{i}-1\right)}^{2}]$	50	[−30,30]	0
$F_{6}(x)=\sum_{i=1}^{n}{\left([x_{i}+0.5]\right)}^{2}$	50	[−100,100]	0
$F_{7}(x)=\sum_{i=1}^{n}ix_{i}^{4}+random[0,1)$	50	[−1.28,1.28]	0

**Table 2:** Multimodal benchmark functions.

Function	Dim	Range	F_min_
$F_{8}(x)=\sum_{i=1}^{n}-x_{i}\sin(\sqrt{\left|x_{i}\right|})$	50	[−500,500]	
$F_{9}(x)=\sum_{i=1}^{n}\left[x_{i}^{2}-10\cos(2\pi x_{i})+10\right]$	50	[−5.12,5.12]	0
$\begin{array}{ll} F_{10}(x)= & -20\exp(-0.2\sqrt{\begin{array}{cc} \frac{1}{n} & \sum_{i=1}^{n}x_{i}^{2} \end{array}})-\\ & \exp\left(\begin{array}{cc} \frac{1}{n} & \sum_{i=1}^{n}\cos(2\pi x_{i}) \end{array}\right)+20+e \end{array}$	50	[−32,32]	0
$F_{11}(x)=\frac{1}{4000}\sum_{i=1}^{n}x_{i}^{2}-\prod_{i=1}^{n}x_{i}^{2}\cos\left(\frac{x_{i}}{\sqrt{i}}\right)+1$	50	[−600,600]	0
$\begin{array}{l} F_{12}(x)=\frac{\pi }{n}\left\{10\sin(\pi y_{1})+\sum_{i=1}^{n-1}{\left(y_{i}-1\right)}^{2}[1+10\sin^{2}(\pi y_{i+1})+{\left(y_{n}-1\right)}^{2}]\right\}\\ \;\;\;\;\;+\sum_{i=1}^{n}u(x_{i},10,100,4)\\ y_{i}=1+\frac{x_{i}+1}{4}\\ u(x_{i},a,k,m)=\left\{\begin{array}{c} k{\left(x_{i}-a\right)}^{m}\begin{array}{cc} & x_{i}>a \end{array}\\ 0\begin{array}{cc} & -a<x_{i}<a \end{array}\\ k{\left(-x_{i}-a\right)}^{m}\begin{array}{cc} & x_{i}<-a \end{array} \end{array}\right. \end{array}$	50	[−50,50]	0
$F_{13}(x)=0.1\left\{\sin^{2}(3\pi x_{1})+\sum_{i=1}^{n}\begin{array}{l} {\left(x_{i}-1\right)}^{2}[1+\sin^{2}(3\pi x_{i}+1)]+\\ {\left(x_{n}-1\right)}^{2}[1+\sin^{2}(2\pi x_{n})] \end{array}\right\}$	50	[−50,50]	0

**Table 3:** Average result of benchmark functions.

F	LSTOA	STOA [30]	PSO [32]	GWO [32]	GA [32]	GSA [32]	SSA [32]
	Ave	Ave	Ave	Ave	Ave	Ave	Ave
F1	8.68 × 10^−18^	2.66 × 10^−17^	9.59 × 10^−6^	6.59 × 10^−28^	5.55 × 10^−1^	2.53 × 10^−16^	1.58 × 10^−7^
F2	2.45 × 10^−12^	6.76 × 10^−12^	2.56 × 10^−2^	7.18 × 10^−17^	5.66 × 10^−3^	5.57 × 10^−2^	2.66
F3	2.13 × 10^−7^	6.26 × 10^−8^	8.23 × 10^1^	3.29 × 10^−6^	8.46 × 10^2^	8.97 × 10^2^	1.71 × 10^3^
F4	1.89 × 10^−4^	2.46 × 10^−5^	4.26	5.61 × 10^−7^	4.56	7.35	1.17 × 10^1^
F5	2.76 × 10^1^	2.77 × 10^1^	9.24 × 10^1^	2.68 × 10^1^	2.68 × 10^2^	6.75 × 10^1^	2.96 × 10^2^
F6	2.40	2.44	8.89 × 10^−6^	8.17 × 10^−1^	5.63 × 10^−1^	2.50 × 10^−16^	1.80 × 10^−7^
F7	2.75 × 10^−3^	1.94 × 10^−3^	2.72 × 10^−2^	2.21 × 10^−3^	4.29 × 10^−2^	8.94 × 10^−2^	1.76 × 10^−1^
F8	−5.47 × 10^3^	−5.39 × 10^3^	−6.08 × 10^3^	−6.12 × 10^3^	−1.05 × 10^4^	−2.82 × 10^3^	−7.46 × 10^3^
F9	8.45 × 10^−1^	2.75	5.28 × 10^1^	3.11 × 10^−1^	3.08 × 10^1^	2.60 × 10^1^	5.84 × 10^1^
F10	2.00 × 10^1^	2.00 × 10^1^	5.01 × 10^−3^	1.06 × 10^−13^	1.64	6.21 × 10^−2^	2.68
F11	1.69 × 10^−2^	9.55 × 10^−3^	2.38 × 10^−2^	4.48 × 10^−3^	5.61 × 10^−1^	2.77 × 10^1^	1.60 × 10^−2^
F12	2.15 × 10^−1^	1.85 × 10^−1^	2.76 × 10^−2^	5.34 × 10^−2^	3.09 × 10^−2^	1.80	6.99
F13	1.62	1.73	7.32 × 10^−3^	6.54 × 10^−1^	3.62 × 10^−1^	8.90	1.59 × 10^1^

**Table 4:** Standard deviation of benchmark functions.

F	LSTOA	STOA [30]	PSO [32]	GWO [32]	GA [32]	GSA [32]	SSA [32]
	SD	SD	SD	SD	SD	SD	SD
F1	2.86 × 10^−34^	9.40 × 10^−33^	3.35 × 10^−5^	1.58 × 10^−28^	1.23	9.67 × 10^−17^	1.71 × 10^−7^
F2	1.32 × 10^−23^	5.89 × 10^−23^	4.60 × 10^−2^	7.28 × 10^−17^	1.44 × 10^−2^	1.94 × 10^−1^	1.67
F3	1.61 × 10^−13^	1.15 × 10^−14^	9.72 × 10^1^	1.61 × 10^−5^	1.61 × 10^2^	3.19 × 10^2^	1.12 × 10^4^
F4	4.82 × 10^−7^	4.57 × 10^−10^	6.77 × 10^−1^	1.04 × 10^−6^	5.92 × 10^−1^	1.74	4.18
F5	4.95 × 10^−1^	5.52 × 10^−1^	7.45 × 10^1^	7.93 × 10^−1^	3.38 × 10^2^	6.22 × 10^1^	5.09 × 10^2^
F6	2.28 × 10^−1^	3.49 × 10^−1^	9.91 × 10^−6^	4.82 × 10^−1^	1.72	1.74 × 10^−16^	3.00 × 10^−7^
F7	4.69 × 10^−6^	3.93 × 10^−6^	8.04 × 10^−3^	2.00 × 10^−3^	5.94 × 10^−3^	4.34 × 10^−2^	6.29 × 10^−2^
F8	1.71 × 10^5^	1.71 × 10^5^	7.55 × 10^2^	9.10 × 10^2^	3.53 × 10^2^	4.93 × 10^2^	7.73 × 10^2^
F9	5.06	6.70 × 10^1^	1.67 × 10^1^	3.52 × 10^−1^	7.57	7.47	2.00 × 10^1^
F10	2.34 × 10^−6^	3.97 × 10^−6^	1.26 × 10^−2^	2.24 × 10^−13^	4.62 × 10^−1^	2.36 × 10^−1^	8.28 × 10^−1^
F11	1.37 × 10^−3^	3.05 × 10^−4^	2.87 × 10^−2^	6.65 × 10^−3^	2.69 × 10^−1^	5.04	1.12 × 10^−2^
F12	2.88 × 10^−2^	5.17 × 10^−3^	5.40 × 10^−2^	2.07 × 10^−2^	4.09 × 10^−2^	9.51 × 10^−1^	4.42
F13	2.61 × 10^−2^	7.23 × 10^−2^	1.05 × 10^−2^	4.47 × 10^−3^	3.10 × 10^−1^	7.13	1.61 × 10^1^

**Table 5:** Coding set size of $A^{GC,NL,UA}(n,d,w)$. where *l* denotes the result for LSTOA, *b* denotes the result for DMVO, and bold denotes the better result in the same case.

N\D	3	4	5	6	7	8	9
4	6 *^b^*						
	**6** *^ l^*						
5	12*^ b^*	5*^ b^*					
	**12** *^ l^*	**5** *^ l^*					
6	30*^ b^*	11*^ b^*	4*^ b^*				
	**30** *^ l^*	**11** *^ l^*	**4** *^ l^*				
7	53*^ b^*	19*^ b^*	6*^ b^*	3*^ b^*			
	**53** *^ l^*	**19** *^ l^*	**6** *^ l^*	**3** *^ l^*			
8	101*^ b^*	38*^ b^*	12*^ b^*	5*^ b^*	3*^ b^*		
	**101** *^ l^*	**41** *^ l^*	12*^ l^*	**5** *^ l^*	**3** *^ l^*		
9	167*^ b^*	58*^ b^*	19*^ b^*	7*^ b^*	3*^ b^*	2*^ b^*	
	**170** *^ l^*	**63** *^ l^*	**19** *^ l^*	**7** *^ l^*	**3** *^ l^*	**2** *^ l^*	
10	250*^ b^*	110*^ b^*	34*^ b^*	11*^ b^*	5*^ b^*	3*^ b^*	2*^ b^*
	**250** *^ l^*	**114** *^ l^*	**37** *^ l^*	**11** *^ l^*	**5** *^ l^*	**3** *^ l^*	**2** *^ l^*

**Table 6:** Comparison of encoding size and time between the DMVO and LSTOA.

		n = 8, d = 3	n = 8, d = 4	n = 9, d = 3	n = 9, d = 4	n = 10, d = 5	n = 10, d = 6
DMVO [25]	Size	101	38	167	58	110	34
	time (min)	22	21.5	23.1	20.9	24	24
LSTOA	Size	101	41	188	63	114	37
	time (min)	19	17.6	20.3	20.5	19.8	20.2

## Data Availability

The data presented in this study are available on request from the corresponding author.
